# A Review of EEG and fMRI Measuring Aesthetic Processing in Visual User Experience Research

**DOI:** 10.1155/2021/2070209

**Published:** 2021-12-16

**Authors:** Zhepeng Rui, Zhenyu Gu

**Affiliations:** Shanghai Jiao Tong University, 1004, NeoBay Building 1, 951 Jianchuan Road, Minhang District, Shanghai, China

## Abstract

In human-computer interaction, the visual interaction of user experience (UX) and user interface (UI) plays an important role in enriching the quality of daily life. The purpose of our study analyzes the use of brain-computer interface (BCI), wearable technology, and functional magnetic resonance imaging (fMRI) to explore the aesthetic processing of visual neural response to UI and UX designs. Specifically, this review aims to understand neuroaesthetic processing knowledge, aesthetic appreciation models, and the ways in which visual brain studies can improve the quality of current and future UI and UX designs. Recent research has found that subjective evaluations of aesthetic appreciation produce different results for objective evaluations of brain research analysis. We applied SWOT analysis and examined the advantages and disadvantages of both evaluation methods. Furthermore, we conducted a traditional literature review on topics pertaining to the use of aesthetic processing knowledge in the visual interaction field in terms of art therapy, information visualization, website or mobile applications, and other interactive platforms. Our main research findings from current studies have helped and motivated researchers and designers to use convincing scientific knowledge of brain event-related potential, electroencephalography, and fMRI to understand aesthetic judgment. The key trend finds that many designers, artists, and engineers use artistic BCI technology in the visual interaction experience. Herein, the scientific methods applied in the aesthetic appreciation to human-computer interface are summarized, and the influence of the latest wearable brain technology on visual interaction design is discussed. Furthermore, current possible research entry points for aesthetics, usability, and creativity in UI and UX designs are explicated. The study results have implications for the visual user experience research domain as well as for interaction industries, which produce interactive projects to improve people's daily lives.

## 1. Introduction

A part of human nature is liking for beautiful things [1]. This idea covers a wide range of disciplines, including human-computer interaction (HCI), which includes hardware and software design and development. HCI is concerned with the design, evaluation, and implementation of interaction and computing systems for human use and the study of the major phenomena surrounding them, such as behavior and psychology demands [2]. Human-computer interface design is more specific, is considered the contact interface between the application and the end user ([3], p.1402), and concerns the user interface.

The main parts of HCI or interface design are the user experience (UX) and user interface (UI) designs, which are related to the neuroaesthetic and aesthetic processing research. With the development of technology innovation globally, people have started to pay attention to user experience in enriching their quality of life. UX [4] is defined as “a person's perceptions and responses resulting from the use or anticipated use of a product, system, or service.” Usually, UX is correlated with human emotion, perception, preferences, reflections, feelings, and psychological variations.

The motivation of this study is to help designers and researchers use brain science technology to understand aesthetic judgment from visual interaction projects. This review article aims to provide an overview of design challenges and their solutions and of problems of discussing subjective aesthetic judgments in the daily design process and visual interaction markets. Moreover, this review summarizes the objective methods of measuring aesthetic appreciation and methods of solving scientific aesthetic judgment problems in visual user experience research. Therefore, the present review aims to analyze human neuroaesthetic processing of UX and UI designs and explore these developments in the visual interaction field.

There are some differences between UX and UI designs, and UI is one of the interaction factors of UX; UX focuses on the entire experience concept or user journey towards a project, whereas UI is related to the visual format, such as animation and color selection [5]. For example, creative artistic therapy brain painting is a type of UX journey [6], but website interface aesthetic design is a type of UI design. The scope of the UX in the present study is focused on visual interaction experience. It covers many fields, such as virtual reality, augmented reality, art therapy, education, game design, public art, UI design, and information visualization. These fields strongly rely on visual content, and aesthetic processing and neuroaesthetics play essential roles here. Furthermore, neuroaesthetics of UX is broader than that of UI; it also involves spatial and temporal effects, environment, virtual space, and social interaction, and it covers more about rhetorical beauty than early first impression visions.

Visual interaction is a critical part of UX, and therefore, neuroaesthetics can contribute to the development of current UX biofeedback. A past brain research review reported that 40% of stimuli concern visual content, 43% of stimuli combine visual and audio, 15% of stimuli contain audio content, and 2% contain others [7]. Neuroaesthetics has been described as a study investigating the neural substrates of aesthetic appreciation [8], and it comprises three aspects: “cognitive neuroscience of aesthetics,” “cognitive neuroscience of art,” and “cognitive neuroscience of beauty.” However, the sense of “beauty” or “aesthetic” is primarily subjective and is a part of neuroaesthetics. As a result, more researchers have started to study neuroaesthetics and analyze the possible methods to determine human aesthetic processing when perceiving everyday interactive things. To test visual biofeedback on beauty, visual stimuli are the first possible method in neuroscience to study the aesthetic feeling biofeedback through event-related potential analysis. Visual stimuli are regarded as the picture or color stimulus presented on a screen, which usually is used to evoke a visual evoked potential [9], containing static and dynamic stimuli. Many researchers utilize visual stimuli to test participants' direct brain activity: for example, P100 is evoked with high color contrast [10] and P200 is sensitive to ugly images [11]. The second method uses time-frequency and topography analysis to study the activation area using electrodes when users observe visual elements. Usually, there are five bands, alpha, beta, theta, gamma, and delta; lower theta is associated with better UX design, and alpha is active with beautiful images [12]. These experimental methods can be implemented using brain-computer interfaces (BCIs), which are communication systems where people interact with external devices solely using brain activity [13]. The third professional method is to use functional magnetic resonance imaging (fMRI) to study brain activation when looking at beautiful objects; the bilateral insular cortex shows a strong response in facially and morally ugly conditions [14]. Event-related potential, time-frequency, and topography methods have been focused on evaluating visual aesthetic processing and can contribute to UX and UI, as discussed in this review article.

The main significance and contributions of this study are as follows:

- Summary of the scientific aesthetic judgment through event-related potential, time-frequency, topography, and fMRI technology
- Finding the potential interactive methods to stimulate visual nerve and enhance the quality of user experience through artistic BCI
- Understanding and mastery of the brain data analysis and technology of aesthetic judgment on visual interaction projects
- Description of the contributions of aesthetic appreciation models related to the visual UX and UI designs
- Discussion of the advantages and disadvantages, limitations, and SWOT (strengths, weaknesses, opportunities, and threats) analysis of subjective and objective evaluations on visual interaction projects

The novelty of this review is that it makes the connection between neuroaesthetics, user experience design, and visual interaction develop the quality of future interactive projects through scientific aesthetic appreciation approaches. Therefore, the present review has two main research questions. What aspects of aesthetic processing affect user interaction and user interface design? How do current researchers use noninvasive brain neurophysiology technology to understand visual biofeedback on UX and UI designs? The following sections explore the research review around these research questions: Section 2 describes the methods for finding relevant papers on aesthetic processing with UI and UX designs.  Section 2.8.3 describes two neuroaesthetic models, top-down and bottom-up analysis.  Section 3 discusses the comprehensive current studies on the uses of event-related potential (ERP), electroencephalograph (EEG), and fMRI for brain region analysis and the data that can aid in understanding the current aesthetic processing of UX and UI designs and in forecasting future developments.  Section 3.11 summarizes all related research papers that developed UX and UI designs, including BCI paradigms, descriptions, participant information, and apparatus. It also involves the SWOT analysis.  Section 4 discusses the advantages and disadvantages, literature comparisons with previous studies, and appropriate usage of brain technology in the visual interaction field.  Section 5 contains conclusions.

## 2. Methodology

The research method of this review is similar to the traditional literature review process. Although the systematic literature review is well defined, it was not used in our research because systematic literature reviews rely on a clear purpose and objective at the beginning of the research [15]. For this review, we read many articles and explored the relationship between brain aesthetic appreciation and human-computer interaction, which requires comprehensive understanding and wide reading knowledge to determine a meaningful topic. The traditional literature review process is creative and appropriate to explore a new topic in a review study and is suitable for a study that has not been strictly defined [16]. After wide reading, we used XMind software to draw a mind mapping figure and find the relationship within the research materials. However, future researchers can use a systematic literature review to explore higher research quality based on this review's research findings.

**Figure 1:**
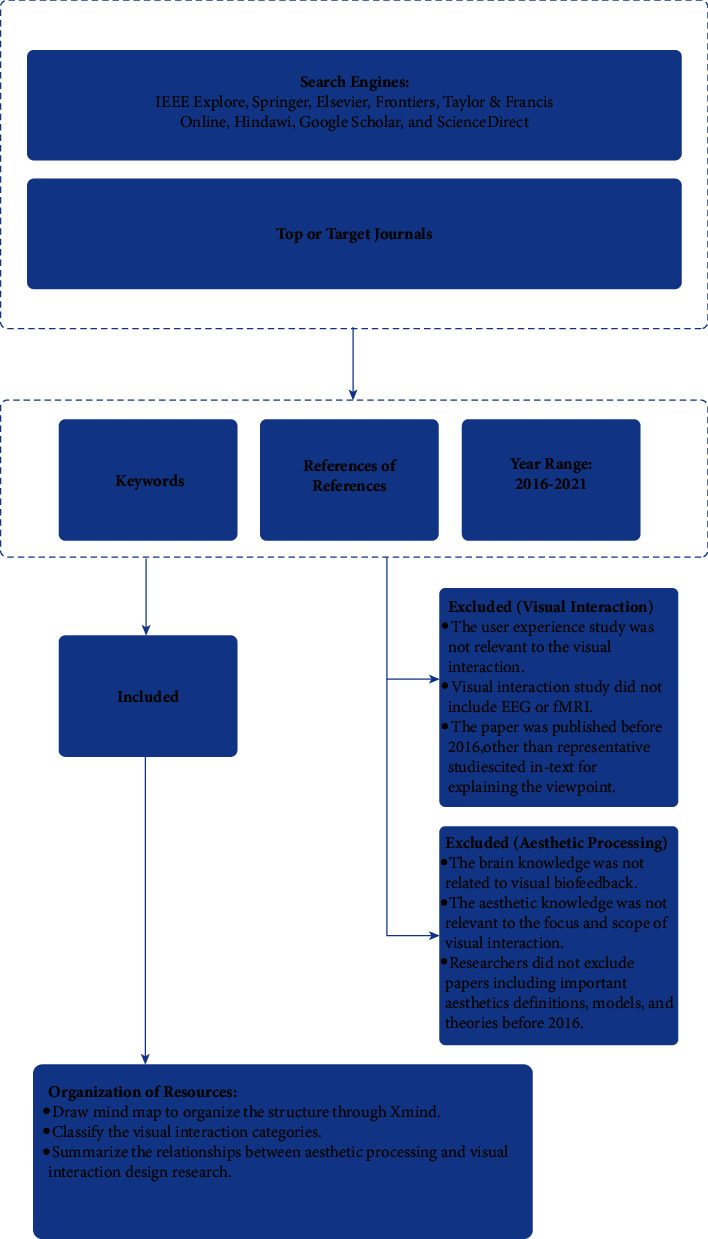
Literature review workflow. The main methods and steps employed in searching, reading, selecting, organizing, and summarizing studies in the literature for this review study are depicted.

The electronic search for research material in this state-of-the-art review was conducted as follows: First, we typed the keywords to collect papers from the following search engines [17]: IEEE Explore, Springer, Elsevier, Frontiers, Taylor & Francis Online, Hindawi, Google Scholar, and ScienceDirect. The keywords used were “neuroaesthetic”/“aesthetic processing”/“brain aesthetic signal processing” + “visual interaction”/“human–computer interaction”/“human–computer interface”/“user experience design”/“user interface design” + “EEG or ERP or fMRI.” The publication year range selected was 2016 to 2021. Second, we also used the same keywords to search several target and top journals: Nature Publishing Group, ACM Transactions on Computer-Human Interaction, Computational Intelligence and Neuroscience, and Interactional Journal of Human-Computer Studies/Interaction. Third, we also searched the References section of retrieved papers because some useful journal articles had cited similar or important papers on visual interaction and aesthetic judgment [16]. The overall literature review research progress is shown in Figure 1. Some representative papers related to aesthetic processing, aesthetic appreciation, and aesthetic judgments published before 2016 were considered, such as those on the topics of classical aesthetic models and neuroaesthetic theories.

### 2.1. Aesthetic Processing in Neuroaesthetics towards UX and UI Designs

The present review article discusses the two most related visual aesthetics models: Chatterjee's model of the neural underpinnings of visual aesthetics and the model of aesthetic appreciation and aesthetic judgment [18]. These two models are the most suitable and related to aesthetic processing on UX design and clearly show the initial, the intermediate, and the overall aesthetic process for designing visual interaction projects.

#### 2.1.1. Model 1: Chatterjee's Model of the Neural Underpinnings of Visual Aesthetics

The first model considers the early vision aspect and concerns the first impression of the UX design. The visual interface activates visual neurons from the frontal lobe to the occipital lobe. The aesthetic experience of a visual artwork begins with a visual analysis of the stimulus, which then undergoes further levels of processing [19]. Meanwhile, attention is normally connected to the frontal lobe associated with cognition, decision-making, and working memory of UX design. In contrast, visual attention has applied visual processing differentially to a focused subset of a scene [20]. It contains overt (gaze attention) and covert visual attention. Visual and cognitive attention is in charge of the complete visual process in Chatterjee's model. Chapman also defined the intermediate vision as “the mechanisms that connect bottom-up early vision with later, task-specific processing.” Intermediate visual reduced retinotopic maps to compact encodings. The intermediate vision can group the selected task-specific early vision components to reflect some preferred representational domain, such as UI, to decide on liking for or wanting of the interface design. The first model is related to UI design, and it concerns early and intermediate vision on aesthetic interfaces and makes a fast reflection of judgment of liking for or wanting of the UI design. Experts and laypersons always consider the following initial visual design principles [21]: affordance, consistency, feedback, visibility, constraints, and mapping. The model also considers the primary step of emotion reflection, but emotional EEG feedback is out of the scope of the present review.

#### 2.1.2. Model 2: Model of Aesthetic Appreciation and Aesthetic Judgment

**Figure 2:**
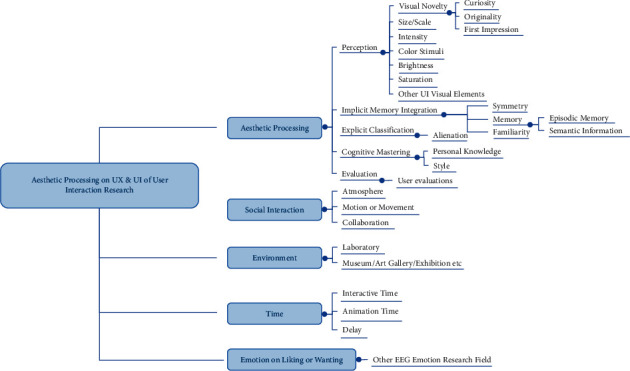
Relationship between aesthetic processing and user interface (UI) and user experience (UX) designs. The structure diagram shows the potential entry points for the aesthetic judgment of user experience research in terms of five main stages of aesthetic processing, spatial or temporal considerations, social interactions or atmosphere, and linking with other emotion studies.

The second aesthetic appreciation model is well known and contains five main steps of aesthetic processing: perception, implicit memory integration, explicit classification, cognitive mastering, and evaluation [22]. The model also includes supplementary factors influencing aesthetic appreciation, such as environment and social interaction. The second model is more concerned with UX because it relates to space or environment, experience consideration, interactive fields, and so on. It includes the overall aesthetic experience of projects. It entails continuous evaluation of the UX design.  Figure 2 summarizes the UX components related to the aesthetic appreciation model (Model 2) [18, 22].

### 2.2. Perception

Visual perception is concerned with the neuro-biofeedback elicited by certain design products, artworks, or art-like stimuli. Visual perception is vivid and full of detailed information, making visual stimuli descriptive, convincing, and persuasive [23]. In the model of aesthetic experience, perception is typically concerned with size, intensity, color stimuli, brightness, and saturation [22]. The initial visual selection follows the salience of objects in the visual field, which can be regarded as a bottom-up approach [24]. Pearson [23] also depicted the bottom-up visual perception as primarily influenced by the eye's recognition of the world.

For example, current research uses the ERP to test aesthetic perception. Aesthetic perception research tried to utilize P100, P200, N200, P300, and LPP to analyze affordance perception and aesthetic neurofeedback [25]. The study attempted to use the high- and low-attractiveness tools to present the oddball process. Especially, P200 represents some aspects of higher order perceptual processing. P100 and P200 show the enhanced amplitude for perceiving highly attractive objects, N200 latency seems to reflect response time, and LPP shows enhanced conditions for unattractive perceptions.

### 2.3. Implicit Memory Integration

UX and visual aesthetic processing rely on certain implicit memory effects. This implicit memory integration is normally in the unconscious state to influence a person's emotional processing. Prototypicality is challenging to measure according to implicit memory integration testing because it depends on a person's individual experience, evaluation, and testing [22]. Implicit memory concerns symmetry and familiarity aspects of the aesthetic stimuli to test the memory inside people's minds. These implicit memory integrations might be related to episodic memory, which influences people's likelihood, attitude, and preference towards objects.

### 2.4. Explicit Classification

Processing is usually affected by the perceiver's profession and knowledge, and explicit classification is meaningful and can be expressed. Interestingly, one recent study found that visual biofeedback differed between expert and layperson perspectives. In one study, the researchers invited ten experts and ten laypersons to judge web page stimuli in an EEG experiment [26]. They found that aesthetic experts needed more time for website evaluation than laypersons. A possible reason is that aesthetic experts have professional knowledge, and they require time for considering evaluation methods and considerations.

### 2.5. Cognitive Mastering

Meanwhile, the ability to reflect further biofeedback and the following stage of cognitive mastering is based on a person's past learning and knowledge. The cognitive mastering results are normally evaluated based on the successful confidence in either revealing a satisfying comprehension, successful cognitive mastering, or changes in the level of ambiguity. “Less is more” mental workload should be considered. In the UX design process, some designers aim to capture user attention and employ very complicated concept designs. However, such a product is less interesting to the end-users because they do not know how and where they should focus their attention owing to the extent of required cognitive information processing. The human body usually delivers 11 million bits per second to the brain for information processing, but the conscious mind seems to be able to execute only 50 bits per second [27]. As a result, designers should put the most important information on the UX design interfaces. For example, researchers revealed that website designers only have 50 milliseconds to capture the users' attention in the website design process [28]. Therefore, more designers follow the design principle of simplicity or design profound concepts in an easy-to-understand method because users will not stay for a long time to understand the interfaces or interactions.

### 2.6. Evaluation

The evaluation stage completes the aesthetic processing through a somewhat more complicated process. In fact, after the explicit stage, participants have already had different notions in their minds. As a result, researchers always invite suitable participants to participate in their studies, such as laypersons, experts, engineers, artists, and designers. Selecting the right participants for brain data analyses directly influences the research outcomes and results. To conduct a user evaluation process on an interactive prototype or project, professional test methods, such as heuristic evaluation, usability testing, and expert evaluations, are used.

### 2.7. Time, Environment, and Social Interaction

Research has found that spatial (environmental) elements have a potential and profound influence on aesthetic processing. Researchers [29] observed that participants spent more time looking at artwork when they viewed it in the context of museums rather than in the laboratory. Researchers have also mentioned “aesthetic attitude” and studied how distance and disinterestedness influence the aesthetic stimuli [30]. This phenomenon highlights the spatial factors that also affect the UX in aesthetic appreciation, affective processing, and usability testing. The research of Herrera-Arcos et al. observed people in a museum to find that beta-band suppression is present during engagement and aesthetic appreciation towards their favorite paintings [31]. Moreover, past research on UX focused on delay and subjectively experienced time. Past studies used fMRI and found that frequent delay causes fewer expectations of future interactive behavior and can activate brain regions such as the posterior medial frontal cortex [32]. The latest research found that subjectively experienced time was in accordance with the perception of interactive temporal characteristics and enhanced UX design [33]. These three supplementary considerations can improve the UX in future interactive research and provide a link to neuroaesthetic analysis.

These two models can contribute to the following discussions and analysis towards UX and UI designs. For example, early human vision is associated with the initial aesthetic network, visual novelty, and first impression when users perceive the UI design. The evaluation stage contains heuristic evaluation user testing for UX quality. Understanding the aesthetic steps from both models would offer research entry points and clarify how aesthetic processing can be involved in the following visual interaction design process.

### 2.8. UX and UI Designs

#### 2.8.1. Creativity

Visual novelty involves visually brand new things that users do not expect and have not experienced before [34]. Kim's research emphasized that visual novelty is an essential factor in pleasure, arousal, expressive aesthetics, classical aesthetics, and perceived usability when the project is perceived initially. There are two words related to visual novelty, creativity and originality. Creative and original works are pieces of work, which are, first, to a significant extent new, original, and distinct and, second, show a high degree of success in the domain [35]. Both creativity and originality can enhance the visual novelty feeling because people like experiencing highly creative projects to improve their “Wow” sense. The frontal lobe and prefrontal cortex play a key role in creativity and help invent more distinctive interactive products [36]. Meanwhile, the past research has found that creative inspiration evokes higher alpha indices than creative elaboration [37]. Therefore, brain status analysis can help further understand the visual novelty of the early vision or perception on UI and UX designs.

#### 2.8.2. Aesthetics and Usability

In usability testing, the functions, features, interactive methods, and perceived usability are the main considerations for evaluating the success of the interactive products. Meanwhile, there are two usability factors: inherent and apparent usability [38]. Inherent usability focuses on the ease of use, and apparent usability is related to aesthetics. In the UX design, usability refers to evaluation by psychological and behavior experience “during and after use (or behavior)” and influences other experience aspects. Another critical term called perceived usability, formed by impression “before use (or behavior),” is affected by quickly formed subjective decisions such as aesthetic judgments and functionalities [39]. On the aspect of UI design, first impression is a part of aesthetic processing. Most user experience contains both elements of aesthetic and usability testing. On the aspect of mobile application, one research utilized N200 and LPP to observe utilization and aesthetic biofeedback of first impressions of mobile applications [40]. This research utilized the brain data analysis to judge the product in four dimensions containing “High usability & High aesthetic,” “High usability & Low aesthetic,” “Low usability & Low aesthetic,” and “Low usability & High aesthetic.” The research found that N200 was higher on the higher aesthetic visual interface and had a lower response to low usability and aesthetic. Contrarily, the low aesthetic can enhance LPP for mobile applications. This research study focuses more on utilizing implicit evaluation. Past research has promoted belief that a pleasant initial aesthetic experience induces the halo effect, mitigating unsatisfying usability problems to affect the overall UX and leading to acceptable design [41]. However, the focus should remain on addressing usability problems. Both UX and UI designs are different from graphic design because UX and UI also need to consider the logical thinking of the design progress.

#### 2.8.3. Top-Down and Bottom-Up Processing on UI and UX Designs

Visual perception, mentioned in both models, is vivid and full of detailed information, making visual stimuli informative and persuasive [23]. It is a process that combines top-down cognition and bottom-up information reception [42]. For the top-down approach, the designer should consider the overall macro design elements, such as the whole design concept of the information architecture. After the initial decision, designers should consider the micro aspects in terms of icon style, design modifications, color enhancement, and other design details. By contrast, bottom-up visual perception is primarily influenced by the eye's recognition of the world [23]. People construct their world view from what they see and observe. On the aspect of UI and UX designs, the bottom-up strategy is used in some uncertain design tasks or unclear main directions. First, designers or researchers find and classify the users' needs in detail and then analyze the corresponding user-centered design directions. Second, designers start to fill up the design gaps and to integrate the overall design situations before finalizing the whole design concept. Designers and researchers are concerned with user behavior and expect to satisfy users' needs, and top-down and bottom-up approaches should complement each other to explore the integrated UI and UX design concepts.

**Figure 3:**
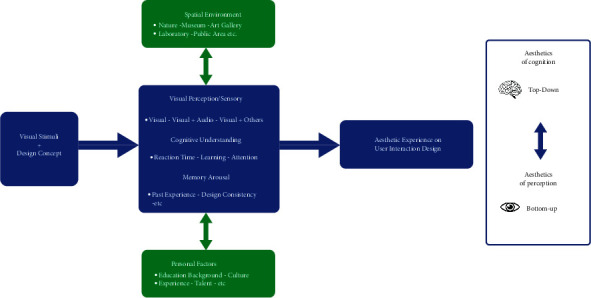
Process of visual interaction and aesthetic processing on user interface (UI) and user experience (UX) projects. The ways in which people appreciate UI and UX projects are revealed. Their visual aesthetic experience is influenced by environmental consideration, design concept, personal conditions, or background through top-down cognition and bottom-up perception.

Top-down and bottom-up approaches also play a role in the selection of visual stimuli and visual information [24]. The initial visual selection is based on the salience of objects present in the visual field. After massive recurrent feedback processing, volitional control based on expectancy and goal setting will bias visual selection in a top-down manner. Top-down knowledge of nonspatial features of the objects in the visual field (e.g., color, shape, and luminance) cannot easily change after the initial selection priority. Most visual information has been determined during the initial aesthetic appreciation, but profound or meaningful content or concepts can alter human perceptions and interpretations. Furthermore, there is an ideal and appropriate model for future HCI invention and creation called “the unifying model of visual aesthetic experience” [43]. The model combines visual and context stimuli to detect human aesthetic experience through bottom-up aesthetic perception and top-down aesthetics of cognition. The model starts with the visual stimulus and content background to construct the basic perception and cognitive thoughts, which is helpful for project evaluation or creation process.  Figure 3 is constructed based on the abovementioned model and involves UI and UX design factors. In general, both top-down and bottom-up pathways are significant methods to explore human visual interaction behavior and the aesthetic appreciation process.

## 3. Current Brain Research on Aesthetic Processing

### 3.1. Experiment Apparatus and Wearable Technology

In the wearable technology area, several companies have invented relatively reliable algorithms to help users know their daily experience through BCI devices such as Muse band, Neurosky Mindwave, and Emotiv EPOC+. These devices have calculated algorithms to output meditation, attention, and emotive reflections when interacting with daily installations, platforms, interfaces, smart vehicles, and other interactive computer applications. The Neurosky Mindwave headset provides meditation and attention values, and Emotiv EPOC+ provides interest, excitement, engagement, stress, relaxation, and focus values. Several studies have used both devices to study visual brain biofeedback on user experience. Past research used a brainwave headset to practice meditation on seven chakras and visualize their minds through Neurosky Mindwave [44]. Antonijevic's team used EPOC+ to test users' visual memory performance [45].

Wearable BCI technology can use data from users' brains and has helped invent many interactive research projects. These reactive and active BCI projects combine artistic design and interactive projects by helping people using art therapy, game design, and education control [46]. For example, the current technology utilizes the P300 BCI to control the preferred game object [47].

Some researchers used 16/32/64/128 electrodes and fMRI to study UX design and aesthetic biofeedback through brain region analysis on the level of clinic apparatus. This apparatus is more advanced and can analyze detailed ERP, topography, time-frequency, and brain region activation. Most EEG apparatus employs the international 10-20 system and has electrodes placed at 10% and 20% points along the lines of latitude and longitude on the head [48]. Meanwhile, past research demonstrated that fMRI could evaluate the topography of the human primary visual cortex [49].

### 3.2. Four Brain Lobes

**Figure 4:**
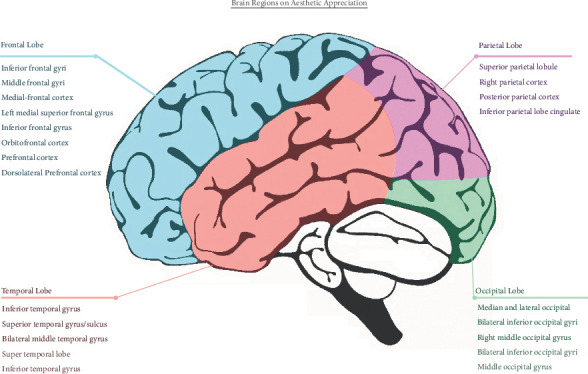
Specific brain regions related to aesthetic appreciation. The critical brain regions are listed under four lobes to help researchers, designers, and artists know their brain research directions to link with the artwork, UX projects, and design materials in developing aesthetic processing research.

To understand the aesthetic reflections and visual stimuli in the context of UI and UX, it is essential to understand the optical functions of brain lobes. The cerebrum contains the frontal, temporal, occipital, and parietal lobes. Each lobe region plays a different role in aesthetic appreciation.  Figure 4 summarizes the areas related to aesthetic processing; these will be updated with future research findings.

#### 3.2.1. Frontal Lobe

The frontal lobe contains inferior, middle, and superior frontal gyri. The frontal lobe [50] relates to attention mechanisms and expression of emotional feelings [51]. The inferior and middle frontal gyri are associated with perceptual, cognitive, emotional, and reward processing feelings. The inferior frontal gyrus and left medial superior frontal gyrus relate to the aesthetic judgments of positive social meanings and beautiful objects. Specifically, the inferior frontal gyrus is also active in the aesthetic senses of rhetorical beauty and moral beauty. The superior frontal gyrus is related to aesthetic judgment and cognitive processing. The orbitofrontal cortex is associated with affective processing. In the spatial environment, it has been found that the medial orbitofrontal cortex and prefrontal cortex have more robust activation in the context of art gallery appreciation than in the digital computer context [52]. The frontal lobe and prefrontal cortex play a key role in creativity and are helpful in the invention of more distinctive interactive products [36]. The frontal area also oversees the formation and manipulation of the mental images and coordinates in the spatial and sensory regions.

#### 3.2.2. Temporal Lobe

The temporal lobe is located between the central sulcus and the parietal fissure. Neurons in the temporal lobe are tuned for 3D surface orientations [53]. The inferior temporal gyrus relates to visual imagery and the representation of object shapes, and the superior temporal gyrus is involved in abstract aesthetic processing [54]. The bilateral middle temporal gyrus and super temporal lobe show reaction to moral ugliness, whereas the inferior temporal gyrus is associated with visual processing and moral goodness [14]. Visual neurons in the temporal lobes have different brain functions.

#### 3.2.3. Occipital Lobe

The occipital regions mainly influence the initial aesthetic network, such as the perception of shape and color [54]. The bilateral inferior occipital gyrus and the right middle occipital gyrus have a more robust reaction to aspects of negative social meaning. The bilateral inferior occipital gyrus especially reflects both ugly objects and negative social meaning. Zhang's team also found that the middle occipital gyrus oversees the appreciation of facial beauty and moral beauty. Occipital lobe visual neurons are more comprehensive in aesthetic processing.

#### 3.2.4. Parietal Lobe

The fourth brain lobe is the parietal lobe. The superior parietal lobule is related to visuospatial exploration [54]. Parietal areas of the scale are also in charge of attractive faces. Moreover, the right parietal cortex [52] shows increased neural activation in response to artistic paintings or images. Past research found that the parietal regions significantly reveal the differences in aesthetic judgment between the sexes [55].

In general, the frontal lobe is the center of reasoning. The parietal lobe is the center of recognition and the perception of stimuli, the temporal lobe is that of visual memories and emotional association, and the occipital lobe is that of visual processing [56]. Researchers [57] have found that the reward circuit in the brain system influences aesthetic appreciation involving the ventral striatum, the hypothalamus, and the orbitofrontal cortex. The reward circuit primarily affects the emotional effects of the UX design, which determines the liking and wanting in the aesthetic models.

### 3.3. Contribution of EEG to UX/UI Design and Aesthetic Processing

The EEG contains the alpha, theta, beta, gamma, and delta bands. The use of EEG can support the observation of a more extended period or epoch than the use of ERP because it supports continuous variation, so it is more suitable in the UX design experiment. Wojciech Salabun described [58] EEG as a measurement of electrical brain activity and state. Azzazy et al. and Bell and Cuevas summarized the EEG as providing direct measurements of electrocortical activity with millisecond precision with high sensitivity to signal changes in arousal, perception, and cognitive process [59, 60].  Table 1 summarizes the contribution of EEG research towards aesthetic processing in the visual interaction.

### 3.4. Contribution of ERP to UX/UI Design and Aesthetic Processing

The event-related potential can detect and record the fast response of the user to the visual, emotional, and usability testing. The event-related potential (ERP) was defined as “a series of voltage oscillations or components recorded from the scalp to indicate the brain's electrical response to discrete stimulus events” [71]. Normally, researchers utilize rapid serial visual presentation to evoke ERP visual biofeedback. Rapid serial visual presentation presents the process of sequential images at the same spatial location at high display rates with multiple stimuli per second [72]. Some researchers applied the oddball paradigm in the experiments. The oddball-paradigm stimuli are classified into two classes: targets and nontargets, and nontargets appear more frequently than targets. The oddball paradigm often contains participants' behavioral responses. As for the specific visual interaction design research, some researchers set aesthetic and usability response questions following every visual stimulus [26, 73].  Table 2 summarizes the contribution of ERP research towards possible aesthetic processing in UI and UX designs.

### 3.5. Contribution of fMRI to Initial and Delayed Aesthetic Appreciation

The human brain contains crucial parts, such as the occipital cortex, which deal with visual information or signals from visual stimulus inputs. These parts of the neuromechanism respond to aesthetic appreciation and are helpful in the interactive visual context. Aesthetic appreciation is related to the activation of two different networks: initial aesthetic network and delayed aesthetic network [86]. People will initially observe the appearance, external morphology, color, shape, and other general appraisals of objects; these are regarded as the initial aesthetic network. Meanwhile, the human sense of beauty is also influenced by other components, such as culture, value, experience, supplementary knowledge, and social appreciation. After deeply analyzing the beauty meaning by consciousness, the delayed aesthetic network emerges in people's minds. For example, in the area of text design, in ancient Chinese culture, abstract art and concrete art have been applied to Chinese text. Research by Wei Zhang's team (2017) focused on the pictographs and oracle bone scripts, which represented abstract social beauty and concrete object beauty, respectively. The study demonstrated that human sense of beauty, ugly, positive, and negative emotions was affected by both object parameters and social values. The research used fMRI to record and analyze the activated brain regions under morphological beauty and social-context positive and negative situations. The research helps explore the direct reflection of both initial and delayed aesthetic considerations from people's minds. These data demonstrate that analyses of neuroscientists can help improve the understanding of both initial and delayed, perceptual and cognitive, aesthetic appreciation for UX improvement. UI design involves the initial aesthetic network, whereas UX design considers both initial and delayed aesthetic networks. In this context, Table 3 summarizes the initial and delayed aesthetic appreciation influenced by object beauty, social environment, culture, background, and context meaning in the human brain regions.

### 3.6. Subjective Evaluations and Brain Analysis towards UX and UI Designs

Some studies show that, in aesthetic preferences, there are significant differences between subjective evaluations and brain data analysis. For example, a UX study on smartphone user experience compared the subjective evaluations and five brain bands of relative power on UX, flow, immersion, controllability, responsiveness, functionality, and pleasure [12]. It showed the significance of correlation of pleasure with beta, responsiveness with alpha, UX with gamma, and immersion and pleasure with theta, which demonstrated that subjective evaluations had some correlations with brain frequency bands but not all of them. In aesthetic evaluation research, Ding's team utilized the ERP to test the visual aesthetic biofeedback on mobile phone product appearance through N100, P200, and N200. Their research found no correlation relationship between P200 and subjective evaluation, which means that brain analysis can reflect more accurate visual preference through electrophysiological data.

Most subjective evaluations of UX and UI designs include heuristic evaluations, probing questions, and user tests. A well-known evaluation model consists of ten principles of heuristics evaluations [87]:

1. The visibility of the system status
2. User control and freedom
3. Error prevention
4. The match between the system and the real world
5. Consistency and standards
6. Recognition rather than recall
7. Flexibility and efficiency of use
8. Aesthetic and minimalist design
9. Help for users to recognize and diagnose errors and to recover from them
10. Help and documentation

Many interactive research experiments have used these ten principles to test users' evaluations on the development of HCI installations and prototypes. These evaluations are quite useful on 2D program applications, but psychophysiological data could be more appropriate for UX or UI designs in the virtual environment [88]. Meanwhile, psychophysiological data are more appropriate for a fast reflection brain system without any user control [89].

**Figure 5:**
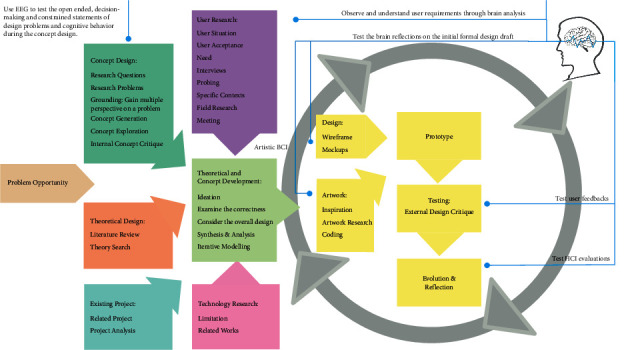
Involvement of brain analysis/experiment in the user experience design process. The design process comprises concept design, implementation, and evaluation methods for UX projects. The possible entry points for brain research in the UX development progress are also shown. By including objective brain analysis, the final effect and performance of UX projects will be more convincing.

In contrast, professional aesthetic judgment should follow the design principles [21], consisting of affordance, consistency, feedback, visibility, constraints, and mapping. These design principles have been applied in many interactive platforms, interface designs, information visualizations, and product designs. However, traditional interview techniques cannot uncover people's unconscious states, and some users even offer unreliable answers that are inconsistent with their consciousness, which influences the quality of research data and results. Daniel Kahneman [89] identified two system models of brain processes. System 1 is fast, automatic, and outside users' control, while System 2 is slow, voluntary, and completely under users' control. Neuroscience brain imaging can detect people's fast reflection system (System 1) to reveal the nonvolitional qualitative data needed to improve visual study reflection and human behavior research in the prototype-testing process. Brain analysis linked to heuristic evaluations and design principles applied in everyday activities should be investigated in future HCI research and applied in the prototype development process. The chart in Figure 5 is inspired by a concept-driven interaction paper [90]. The chart combines the brain analysis and the HCI workflow through the accumulated experience on interaction design implementation. The graph describes the potential BCI utilization during the HCI-developing process. The variety of BCI data categories is beneficial for improving design idea creation, extensions, user evaluations, and prototype development. The variety of brain data categories and status analysis helps improve HCI idea creation, extensions, user evaluations, and prototype development [91].

### 3.7. Neuroaesthetic and Artistic BCI UX Projects

Excluding experimental research studies on aesthetic processing and UX, many past and recent projects have started to connect neuroaesthetics and UX to develop interactive visual installations, projects, and products. Meanwhile, artistic BCI can help record people's brain data and understand users' affective states through creative forms [46]. Visual interaction of artistic BCI consists of passive, active, and reactive BCIs. Active and reactive BCIs are especially applicable because they control the interactive outputs such as artwork independently or dependently of external events through users' biofeedback on the interactive interface. This technique can be utilized to combine with mindfulness, health benefits, education, game, market promotion, and many interdisciplinary fields.

Currently, the BCI can record users' meditation and attention values to analyze their current mental status. The meditation value can test the mindfulness effect of the corresponding art therapy [44]. Neurosky Company developed one wearable technology called the Mindwave headset, and the company invented meditation algorithms. The meditation algorithm indicates the level of mental relaxation, and the value ranges from 0 to 100, which helps users know their inner state of mindfulness. In contrast, attention algorithms indicate mental focus and familiarity and measure learning and understanding with educational tasks [92]. The latest review described that the meditation or relaxation state would cause stronger theta and alpha and weaker beta waves [93]. Furthermore, stronger beta rhythms and weaker alpha and theta rhythms were observed when the user was in a concentration or attention state. Both attention and familiarity data help manage and recognize people's learning effectiveness through BCI technology.

Mindfulness can help people reduce stress and enhance cognition. The concept of mindfulness originated in Buddhist psychology and has been well documented in clinical interventions [94]. Medical doctor Jon Kabat-Zinn from the Massachusetts Institute of Technology created the Mindfulness-Based Stress Reduction program [95], which targets stress reduction, improves heart/body/mind health, and enhances immunity. Current wearable BCI technology can link with mindfulness and user meditation data to create a brain-painting artwork. Mindfulness art therapy is combined with seven-chakra meditation to improve the mindfulness effect through digital visual stimuli technology [44]. Pauline Wills [96] classified chakra concepts into seven colors: red-base chakra, orange-sacral chakra, yellow-solar plexus chakra, green-heart chakra, blue-throat chakra, indigo-brow chakra, and violet-crown chakra. Past research used abstract brain wave patterns to depict the seven-chakra situations to help users visualize their current meditation mind status [44]. Abstract art brainwave patterns intensify people's interest in practicing healthy mindfulness exercises. The combination of visual art and mindfulness can refine users' emotions and feelings. Positive emotions can improve cognitive processing and enhance memory ability and decision-making [25]. Therefore, the combination of visual stimuli and mindfulness has been expected to reduce people's stress, recover emotions, and enhance cognitive abilities. This research utilizes the BCI to combine the visual aesthetic and user experience designs.

Both concrete and abstract art can contribute to UX and UI research of artistic BCI interaction. Abstract art breaks away from the traditional representation of physical objects [97]. In contrast, concrete art makes people think directly about real things without fabrication [98]. Visual interaction derives from the graphical UI (GUI) in the BCI. With digital and computer technology development, visual artists can draw and paint their artworks using the GUI by linking it with a drawing board. Visual interaction also gives rise to digital innovation in abstract and concrete art through the BCI. The University of Sydney Design Lab invented BCI public art projects on the digital canvas in Central Park [99]. Master students presented their digital drawings, videos, and interactive animations on the digital wall by inputting users' meditation, attention, delta, theta, alpha, beta, and gamma values. This project considered the overall environment immersion and presented people with creative ideas on the digital interface. In contrast, recent research [100] created a human-machine interface platform to draw public painting through robotic machines and P300 ERP values. The current wearable BCI technology and human-machine interface have been explored and have demonstrated how the BCI supports HCI, neurophysiology, art, and computing.

Some UX/UI designers used the concept of describing abstract presentations to the public. If an ambiguous interactive abstract art idea is presented, people may not understand the abstract user interface promotion. In research experiments, the BCI also helps push the interpretation of abstract art. The ERP N400 was used to study understanding and meaning in the abstract art context, and the research found that N400 can test “related” or “unrelated” meanings in abstract art [84]. The study revealed that users could perceive and link related context with the corresponding abstract artwork through N400 data analysis. Unrelated contexts elicited more negative N400 amplitudes than related ones. This finding encourages and motivates UX/UI designers to create and invent more interactive abstract art installations and products. Sometimes, abstract content can leave distinct impressions on customers.

### 3.8. Visual Memory Research

To investigate visual memory on UI design, many researchers considered the affordance and consistency of the design progress. In the study of brain, researchers found that the ventral and dorsal pathways oversee the perception of objects and spaces formed in the episodic memory, which is defined as explicit memory of specific items or events tied to a particular spatiotemporal context [53]. Recent UX research of a navigation system [101] in the virtual environment investigated environmental visual stimuli. Users are needed to recognize and memorize the spatial content and structure in virtual game, learning, and exercising domains. The study of dorsal and ventral pathways can help understand brain data used to enhance spatial and temporal HCI experiences. According to Connor and Knierim, the hippocampus should also be considered in this research [53]. They stated that the binding of objects to locations takes place in the hippocampus representing spatial and temporal information, which builds human episodic memory. It oversees the object-space and spatial-temporal navigation systems. Moreover, the hippocampus organizes some milestones and landmarks in people's minds, and it works with ventral and dorsal pathways. Future BCI electrodes and research experiments should concentrate on the hippocampus to study episodic context reflections. The current technology had explored the method to synchronize the GUI with EEG signal measurements to test intentional and incidental memory evaluation on visual memory [102].

### 3.9. Interactive Information Visualization

User experience and user interface receive information and data from GUI, and many designers and artists started to contribute their creative ideas of designing information or data visualization interfaces to improve the UX design in terms of multiblock data analysis, pattern animations, interactive platforms, virtual reality, finger movements, and so on [103, 104]. Information visualization presents interactive visual representation of data to amplify cognition and enhance information understanding [105]. It creates meaningful interfaces to communicate ideas or facts about data and to explore the discoveries.

Recent research has found that dynamic visual stimuli are considered more beautiful and visually pleasing than static visual stimuli [1]. Animated visual stimuli are more interesting to read and interact with than a static UI. Animated data or information visualizations can enhance the acceptance and interest in reading data or information. Research has found that animated visualization can engage users and facilitate learning [106]. Furthermore, the current UI animation time research of Skytskyi [107] found that the most optimal animation time is around 200–500 ms. It suggests that future research can use the animated UI to improve event-related potential studies because the duration of visual stimuli is always less than 1 second in ERP experiments.

In their recent research, Nuamah's team used the EEG to analyze people's frontal and parietal areas to determine the cognitive reflection of information visualization [108]. They found that visualization of inherently spatial (intuitive) tasks reduces cognitive mental work and effort when it is in a spatial or graphical condition than symbolic or numerical conditions. This finding can help UX design of memory and cognitive effects in reading data information. The latest research has also demonstrated that the use of EEG, eye movement, and visualization logs showed the significance for current and future visualization evaluation [109].

Meanwhile, BCI data and information have been used in the interdisciplinary research fields of health, gaming, education, and entertainment. Information and data designs apply to BCI information presentation. When designing BCI information for healthcare, it has been suggested that the design should follow rules that are thoughtful and clear [110]. The rules produce a specific characteristic or pattern of action and encourage moral, physical, and mental development in a particular direction. The visual stimuli of the information design should be acceptable and convincing.

In other interactive fields, pictographic symbols have become essential components of the systems. These symbols are used to indicate information to people through visual design hints. Pictographic symbols appear in people's daily interactions: in mobile applications, web pages, interactive game installations, shopping mall navigation systems, and so on. Illustrative and legible icon designs allow people to quickly comprehend the meaning of the interface and navigate their interactions accordingly. More specifically, color usage is widely applied in users' interactions. BCI research [10] reported that color selection can play an essential role in pictographic design. P100 is extremely sensitive to high-preference color combinations (blue and white), which enhances the level of legibility. P300 can test the attention and reaction times for icon design legibility, and the mean amplitude for shorter icon display times was significantly higher than that for longer icon display times. Brain research can contribute to the color and legibility context in the design of pictographic symbols in daily applications.

The colors also connect with people's emotions and become the designers' inspiration to create a color interactive installation to refine people's daily feelings. Ambient BCI color design should focus on the users' moods and consider the use of artistic patterns to improve people's everyday environment. Research has found that a combination of colors and emotions resulted in significantly higher classification accuracy and evoked stronger P300 and N400 reflections than a gray face pattern and color ball pattern [111]. This research demonstrates that affective, meaningful patterns and color can enhance people's visual impressions and evoke higher ERP values.

Current data visualization is also a part of UX and UI in future research development. The professional data visualization book classifies the information into Data Process, Data Blocks, Data Circles, Data Log, Data Nets, Data Maps, and Data Aesthetic [112], which improves the UX and UI designs in many interdisciplinary data categories. Future neuroaesthetic research towards UX and UI improvements on information visualization could refer and obtain inspiration from Data Flow book on understanding data and further aesthetic processing improvement through EEG and ERP analysis.

### 3.10. Influence of Brain Impairment Conditions on UX and UI Designs

Many researchers recruit participants to join their brain or eye test experiments. However, the conditions in Table 4 should be considered when testing user interaction on UX and UI designs using aesthetic processing and visual biofeedback. This summary of brain impairment conditions can inspire UX designers to invent more interactive installations or products to help visually impaired people understand this world. For example, current studies utilized visual-to-auditory sensory substitution devices to convert visual information to auditory information [113], and Mymemory mobile memory application helps people with traumatic brain injury [114]. Table 4 summarizes brain impairment conditions that influence aesthetic appreciation and UX and UI designs.

### 3.11. Comprehensive Summary of Current Trends and Opportunities in UI, UX, and Aesthetic Processing

Some environment considerations are not directly related to human-computer interaction but are suitable for considering future user experience design in human-computer installations.  Table 5 summarizes the recent research studies contributing to the UI and UX designs.

Table 5 summarizes the UI and UX visual stimuli/content related to brain research. The table is ordered alphabetically based on visual stimuli/content. By understanding Table 5, researchers can understand current and future research developments in the area.

After reviewing current brain region analyses on aesthetic processing, it is clear that researchers have used different types of visual stimuli (interface, interactive product shape, animation, information visualization, etc.) and brain neurophysiological methods to analyze the aesthetic biofeedback in the UI, UX, and interactive outputs. The SWOT analysis in Table 6 describes brain research on aesthetic processing contributing to UX and UI designs, which is summarized according to noninvasive neurophysiology SWOT research [151].

## 4. Discussion

The main reason for conducting this review was the lack of research review concerning brain aesthetic processing of the visual interaction towards user experience design. Our paper can contribute to better understanding of the ways researchers and designers can use EEG and fMRI to analyze the aesthetic judgment in user interface and user experience designs through ERP, time-frequency, topographies, and fMRI data analysis. In this study, the research findings and results demonstrated that every ERP amplitude could contribute to visual interaction, projects, and analysis. Then, the activated or inactivated status of time-frequency, topography, and fMRI can provide understanding of users' aesthetic biofeedback on the visual interface. Finally, many designers and engineers started to import EEG data into the daily visual interaction applications.

### 4.1. Advantages and Disadvantages

An objective evaluation has some essential advantages and disadvantages when developing visual interaction design. The main advantage of an objective assessment of aesthetic appreciation it that it is convincing and supported by scientific knowledge. The objective evaluation covered users' unconscious status without any user control. It is connected to visual neurology of human physiological development and evolution. The advantages of the review study also can support the emotional brain systems to develop current and future intelligent visual interaction systems. Other than the limitations and weaknesses described in the SWOT analysis, the main disadvantage of recent objective evaluation is the difficulty of working with open-ended questions in aesthetic preference. All the visual stimuli reported on were designed in a restricted way by researchers and designers. At this time, subjective evaluation should be included in studies. The user experience researcher should probe and ask open-ended questions about developing an interface design and preferred direction by recording participants' answers. It is reasonable to combine both subjective and objective evaluations for future visual interaction in user experience research.

### 4.2. Visual and Emotional Orientations

Recently, review research has primarily focused on the relationship between emotions and EEG studies [7, 17]. They discussed how valence and arousal influence the user experience quality and summarized the corresponding activated brain regions for anger, disgust, fear, happiness, and sadness. Meanwhile, emotion-related studies used the wearable Emotiv EPOC+ headset to understand emotion status during the user experience process. Furthermore, an interesting review study presented similar findings to user experience research from EEG and EMG studies [16]. The work discussed professional studies on emotional biofeedback in terms of game context, usability testing, and applications through EEG study. It also discussed wearable technology in the gaming environment and collected emotional EEG data and information. However, these studies did not mention aesthetic appreciation of visual user experience design. The key difference from our review study is that our study shows a clear influence of aesthetic processing on visual interaction research. Our study created a new connection between aesthetic appreciation, brain data analysis, and visual interaction design, which helps researchers and designers with the use of scientific aesthetic judgments for daily interactive product or interface design. Furthermore, this review considered the ways wearable EEG headsets can enhance the visual interactive user experience in people's everyday lives, such as brain painting and visual memory research. The study discussed the input/output brain data in daily interactive art therapy platforms. Through active or reactive BCI data input/output, people can visually interact with an intelligent system to enhance user experience design. Meanwhile, our study can support emotion-related applications and allow more users to accept daily BCI interactive applications. Past research stated that positive emotions improve cognitive processing, visual sensory perception, attentional resources, memory ability, and decision-making [25]. Our research results can help researchers combine visual and emotional research orientations in visual interaction and the user experience market. The two aforementioned aesthetic models can combine both visual and emotional orientations for future user experience studies.

### 4.3. Appropriate Use of Brain Technology in Visual Interaction

In this review, researchers recommended the ERP, EEG, and fMRI to measure aesthetic judgment of daily interactive projects. First, researchers can use the ERP to present the picture stimuli or short film (1 to 2 seconds) and observe fast visual biofeedback towards these visual stimuli, such as visual animation and website user interface [26]. Second, researchers can use time-frequency to observe a longer period of user interaction behavior and compare these brain data with the rest-mind status [152]. Third, the topography and fMRI can detect higher or lower activation for specific brain regions. Past research found that relative power of EEG analysis (delta, theta, alpha, beta, and gamma) was quite useful in analyzing activation of brain regions during smartphone user experience [12]. Fourth, researchers can allow participants to use wearable headsets to record data outside the laboratory [31]. Meanwhile, artistic active or reactive BCI can import users' EEG data and present creative forms on the visual interaction application. These inventions can enhance the original user experience quality, such as mindfulness brain painting [44]. Finally, future researchers and designers can combine computational intelligence (machine learning, deep learning, etc.) and brain neuroscience technology to discriminate visual interface images. For example, the relevant review described that feeding calculated network measures into support vector machines and using P300-based brain network analysis could improve the classification accuracy of visual stimuli [118]. Through the appropriate analysis of brain data, researchers can improve more interaction platforms.

### 4.4. Limitations

The primary limitations of the psychophysiological measurements of price, complexity, environment consideration, device format, time consumption, data dependency, physical movements, noise interference, and knowledge accumulation should also be considered [16] before the physiological experiments are executed in this visual user experience research field.

Our study also found that using objective brain evaluations poses a difficulty in exploring open questions from users' assessments in the visual interaction context. Users' communications and opinions towards aesthetic appreciation are limited in the experimental physiology resources and processes. Moreover, owing to the high cost of physiology experiments and complex process of data analysis compared with experiments involving subjective evaluation, the number of participants must always be considered for EEG and fMRI experiments.

Finally, the limitation of this study is the lack of analysis connecting aesthetic appreciation and emotion. The research focuses on the visual interaction with UX and UI designs and does not contain other sensory interactions. Thus, a limitation of the study is that it only focuses on brain science and data analysis, and future research should also involve detailed analysis of combinations of other psychology measurements to study aesthetic judgment, for example, with the use of an eye tracker.

## 5. Conclusions

In this review, we have described the methods that use EEG and fMRI to analyze aesthetic processing of visual interactions in user experience research. Many researchers have analyzed brain data to obtain neuroaesthetic biofeedback on UX design in terms of website, mobile application interface, art therapy, brain painting, vehicle human-machine interface, virtual or augmented reality, information visualization, and so on. The critical importance of the study is that it has summarized the corresponding event-related potential and brain region activations when perceiving the visual interaction projects. Another critical point is to motivate people to apply artistic BCI applications in daily visual interactive experience. Designers and engineers can combine both creative and logical ideas to invent more active BCI interactive applications. Understanding the aesthetic appreciation models will clarify the appropriate methods to analyze the aesthetics and usability in the visual interaction development process. Therefore, the study has benefits for researchers, designers, and engineers who use brain scientific research results to support aesthetic appreciation preference. Furthermore, this review encourages UX and UI researchers to combine subjective and objective evaluations to demonstrate the research results for the appropriate interaction context. The implications of the review are mainly for visual interactions in the user experience research domain and users who want to pursue a better aesthetic user experience to enrich their life quality. In summary, this paper provided a helpful review updated with the current research in aesthetic appreciation, models, EEG, and fMRI in the visual interaction area; these offer a new neural aesthetic research base for future user experience research.

## Figures and Tables

**Table 1:** EEG contributions to UX and UI aesthetic designs.

Wave name	Frequency (Hz)	Typical amplitude (uV)	Meaning	Aesthetics on UX	Aesthetics on UI
Delta	0.1–4	100–200	The deepest stages of sleep and support in describing the depth of sleep	Better UX evokes stronger relative power of delta (frontal region) [12].	Delta reflects feelings of drowsiness, which can be used to evaluate the visual novelty of the UI [61, 62]. Frontal delta is related to the visual working memory, which can be used to evaluate the complexity of UI design [63].
Theta	4–8	Higher than 30	Interesting emotions, distraction, trance, hypnosis, and intense dreams and emotions	Better UX affects weaker relative power on the theta rhythms (frontal and parieto-occipital regions) [12]. Theta is correlated with UX on navigation and location recall, which can be considered in the virtual environment [64].	Preferred humanoid robot appearances showed higher theta rhythm power than nonpreferred ones [65].
Alpha	8–13	30–50 or higher	Relaxed but aware state	Better UX evokes stronger relative power of alpha (frontocentral, parietal, and parieto-occipital regions) [12]. EEG asymmetry of the alpha frequency band is strongly associated with affective or emotional processes [66]. The alpha power varies the function in tasks related to creativity and interventions [18].	Alpha brain waves are active when people feel stable and pleasant [51]. Alpha increased when users were in a relaxed state or showed pleasing images [12]. Posterior alpha is in charge of visual working memory, which can test the complexity of UI [63].
Beta	13–30	2–20 or higher	Excited state, physical activity	The beta frequency band is associated with emotions in the immersive or virtual environments [67].	Studies reported that women showed higher beta in the anterior cingulate cortex on modelling design [68].
Gamma	30–60	3–5 or higher	High consciousness level, integration of different sensory modalities, awakened state	Better UX evokes stronger relative power of gamma (C3) [69].	It is in charge of working memory of graphic UI design [70].

UX, user experience; EEG, electroencephalograph; UI, user interface.

**Table 2:** ERP contributions to UX and UI aesthetic designs.

ERP	Amplitude	Contribution of ERP to UI and UX designs
**C1**	(Positivity or negativity) 25–125 ms	C1 reflects the visual activity and attention effects in V1 [74].
**P100**	(Positive wave) 50–150 ms	P100 results from early ERP amplitude at the occipital lobe [10]. Some research found it reflected early visuoperceptual processes mediated by attention. P100 is extremely sensitive to high contrast color combination, which is suitable for user experience design research [10].
**P200**	(Positive wave) 150–250 ms	It reveals a P200 amplitude increase in response to ugly images, which was probably the result of a negative bias in attentional processes [11, 75]. It is also sensitive to negative emotional pictures. P200 is also the charge of the design affordance [76].
**P300**	(Positive wave) 250–350 ms	The P300 peak is shorter for color stimuli [77]. It reflects the distribution of attention and decision-making [25]. It is more positive for consistent and beautiful faces [50]. On the contrary, P300 is also used to explore emotion processing of first impression between emotion and attention [69].
**N100**	(Negative wave) 80–120 ms	N100 is associated not only with the physical features in reflection to the attention level but also with the attractiveness of stimuli [78]. It relates to package design.
**N170**	(Negative wave) 120–200 ms	N170 is the ideal ERP component for facial identification and attractiveness [79, 80]. Happy emotion elicited larger N170 amplitudes [81].
**N200**	(Negative wave) 200–350 ms	N200 shows the negative component peaking at 200–350 ms. It reveals the perception of beauty, especially of geometric shapes [69].
**N300**	(Negative wave) 270–400 ms	N300 is responsive to picture stimuli and affordance [82, 83].
**N400**	(Negative wave) 350–500 ms	N400 activates between poststimulus onset with a frontal-central to central-parietal scalp distribution. N400 is concerned with the measure of semantic processing of words or pictures [83, 84].
**N450**	(Negative wave) 380–530 ms	Attractive user interface design evokes more negative amplitudes, which shows a larger N450 [73].
**LPP**	(Late positive wave) 550–770 ms	Late positive potential mainly oversees delayed aesthetic perception and understanding of the objects [85]. LPP shows a larger response to low aesthetic interfaces than to high aesthetic ones [40].
**VPP**	(Positive wave) 160–200 ms	Facial stimulus and representing the stage of face structural encoding [80]. It is associated with the visual memory processing because it is closer to the hippocampus.

UX, user experience; EEG, electroencephalograph; UI, user interface; ERP, event-related potential.

**Table 3:** fMRI findings on initial and delayed aesthetic appreciation.

Aesthetic status of UX and UI designs	Brain regions
Aesthetic judgments of beautiful object morphology *(initial aesthetic appreciation)*	Bilateral inferior occipital gyrus, left middle occipital gyrus, bilateral inferior frontal gyrus, left medial superior frontal gyrus, right inferior OFC, left hippocampus, left superior parietal lobule, right supramarginal gyrus extending to the postcentral gyrus, and right paracentral lobule [54].
Aesthetic judgments of rhetorical beauty *(delayed aesthetic appreciation)*	Bilateral inferior occipital gyri and inferior frontal gyri, left medial superior frontal gyrus, bilateral hippocampus, and right putamen [54].
Aesthetic judgments of both beautiful object morphology and rhetorical beauty *(initial and delayed aesthetic appreciation)*	Bilateral inferior occipital gyrus, left middle occipital gyrus, bilateral inferior frontal gyrus, left medial superior frontal gyrus, left hippocampus, and right inferior OFC [54].
Ugly and negative feelings on object morphology and rhetorical description *(initial and delayed aesthetic appreciation)*	Bilateral inferior occipital gyri during judgments of pictographs that referred to ugly objects and oracle bone scripts that referred to negative social meaning [54]. The bilateral insular showed the largest response in the facial ugly and moral ugly conditions [14].

fMRI, functional magnetic resonance imaging; OFC, orbitofrontal cortex; UX, user experience; UI, user interface.

**Table 4:** Brain impairment influence on aesthetic processing of UX and UI designs.

Impairment condition	Influence on aesthetic appreciation	Influence on UX and UI designs
Stroke	1. Change of the dominant hand to create artwork [115]. 2. Negatively affects the emotions in response to the music if the insula was impaired [116].	1. Influences the user control hand behavior on user experience. 2. Interactive visual and audio art therapy may reduce the quality of user experience.
Visual agnosia	Patients cannot render the overall forms of the objects, but only some essential features. Impaired artists cannot draw from memory but rely on copying objects from the real world [116].	Influences design affordance experience of UI design because of impaired visual memory.
Degenerative disease: Alzheimer's disease	1. Some patients have frontotemporal dementia (semantic dementia), which influences new interests in art. They paint some repetitive painting patterns [116]. 2. Artists keep their original art preferences, the same they had when they were healthy [117]. 3. A recent review found that patients with Alzheimer's disease influence the clustering coefficient in the right occipital electrode, and the topology of the brain network is altered [118].	1. Influences the visual novelty experience on receiving new UX and UI designs. 2. Influences the results of complex networks and deep learning on connecting the EEG signal and visual elements' classification results.
Epilepsy and migraine	Influence the variety of visual elements and inspiration [117].	Influence the results of a variety of visual stimuli on the event-related potential.
Left temporal lobe resection because of epilepsy	Changes in the preference for music, artworks, and literature [119].	Influences the results of subjective evaluations or online questionnaire results.
Damage to the amygdala	1. Higher liking for three-dimensional visual stimuli of geometrical shapes [120]. 2. Easier recognition of music features in scary/sad music than in happy music [121].	The graphic design interface was affected because of preference for three-dimensional visual stimuli.
Posttraumatic disorders	Patients find it difficult to achieve high accuracy because of the need to pay attention continuously [77].	Influence high concentration research using event-related potential or fMRI.
The impairment of eye or brain visual systems	1. The gaze-independent hybrid-BCI experiment is not quite effective [122]. 2. It loses navigation or motion ability in a varying spatial environment, but it can help computer-aided design on environment research [123].	1. Possibly influences many research studies on aesthetic processing. 2. Influences the navigation research of UX or UI designs.
Lack of access from perceptual system such as specific musical anhedonia	Reduces emotional pleasure from music and visual art. Affects the hedonic sensory system [57].	Influences the perception step of aesthetic processing of UI design.
Motion sickness	Motion sickness influences BCI accuracy in virtual reality-based applications [47].	Influences UX in virtual environment research studies.
Mental and visual fatigue	Mental fatigue negatively influences users' affective experience with the visual stimuli test [7]. Visual fatigue will cause participants to feel tired, which will affect electroencephalograph and event-related potential data accuracy and experiment results.	Influences the results of event-related potential and fMRI research on UX design. Some researchers started to use low visual fatigue and low contrast to present visual stimuli to avoid visual fatigue [124].
Traumatic brain injury	1. Frontal and prefrontal areas of the brain are impaired [125]. 2. Cognitive communication impairments and difficulties.	Influences the visual memory test on memorizing the UI information or data.
The limitation of dorsal and ventral streams	1. Disparity between virtual and real worlds. 2. Conflicting visual depth information.	Virtual reality needs to consider dorsal and ventral functions in perceiving real life [126]. There are many differences between virtual and real environments.
Autism spectrum disorder	1. Has an impairment of empathic ability, which is related to aesthetic perception [127].	Influences implicit and explicit evaluations of aesthetic perceptions.

fMRI, functional magnetic resonance imaging; BCI, brain-computer interface; UX, user experience; UI, user interface.

**Table 5:** Comprehensive summary of all current brain aesthetic processing research studies on UX and UI designs.

References	Visual stimuli/content	UI/UX categories	Number of participants	BCI apparatus	BCI paradigm, electrodes, and brain area	Contributions to UI/UX
[44]	Brain painting	UI and UX	42 (questionnaire)	Neurosky Mindwave	Active BCI on FP1 position	The research created brain painting and used the seven-chakra meditation concept. The mindfulness meditation UX and creative UI helped people decrease stress.
[100]	Brain painting	UI and UX	681 (questionnaire)	Wireless g.Nautilus	P300 BCI with electrodes Fz, Cz, P3, Pz, P4, PO7, Oz, PO8	Used P300 BCI and robotic machine to draw public art painting in a public area. It is a representative study of public art UX design.
[128]	Brain painting	UX	8	16 dry-electrode channels with the G Tec Nautilus EEG device	P300 BCI with electrodes Cz, CPz, P1, P3, P5, P7, Pz, P2, P4, P6, P8, PO3, PO7, POz, PO6, and PO4	The study helped people with amyotrophic lateral sclerosis experience brain painting through P300 BCI with VR installations.
[77]	Color, shape, and animation	UI	37	Monopolar 25-channel EEG with linked earlobes reference using an NVX-52 amplifier	RSVP paradigm P300-based BCI recorded in the O1, PO3, PO7, and T6 sites and the factor of color stimuli on O1, O2, PO3, and PO4. PO8 and POz sites were also affected	P300-based BCI works accurately with color stimuli and secondarily with shape stimuli.
[68]	Color and environment	UX	30	US Neuroscan EEG recording and analysis system with 64-channel electrodes	Event-related spectral perturbation to observe the theta (4–7 Hz), alpha (8–13 Hz), beta (14–30 Hz), low gamma (31–50 Hz), and high gamma (51–100 Hz) bands	The research considered the UX environment of the interior design inside a driven car. Men and women showed different brain high-gamma and high-beta feedback based on the color tone of the interiors.
[1]	Dynamic and static landscape	UI and UX	22	fMRI	Brain feedback on the regions of occipital lobe, frontal lobe, supplementary motor area, cingulate cortex, insula, middle temporal gyrus, and hippocampus	The research study demonstrated that dynamic visual stimuli were more visually pleasing than static visual stimuli by comparing and analyzing static and dynamic landscape stimuli.
[111]	Emoji design	UI	10	g.USBamp and g.EEGcap	RSVP paradigm; gaze-independent BCI and event-related potential on the following electrodes: Cz, Pz, Oz, Fz, F3, F4, C3, C4, P3, P4, P7, P8, O1, and O2	Participants evoked P300 and P400 amplitudes on colored dummy faces.
[129]	Environment consideration of multisensory perception	UX	24	64 electrodes	Focus on alpha, beta, and gamma. P100, N100, P200, N200, and P300 were selected Details of the electrodes are described in the article	Research to help determine the combination of multisensory perceptions of UX.
[130]	Environment consideration	UX	20	64 silver (Ag/AgCl sintered) electrodes on a stretch Lycra Quik-Cap	EEG on alpha	The study compared personal aesthetic and affective responses towards paintings and public areas of commercial stimuli. It suggests the public UI and UX designs have a far-reaching influence on people's decision-making.
[31]	Environment consideration	UX	209	Muse band (MoBI)	AF7, AF8, TP9, TP10	The research considered the environment on perceiving artwork, which can be used for future public interactive art such as TeamLab.
[71]	Game interfaces and interactions	UI and UX	-	-	-	The paper summarizes the comprehensive EEG, ERP, and SSVEP brain analysis on game UI and UX designs.
[131]	Icons (graph and text)	UI	25	Australian Compumedics Neuroscan 64 EEG acquisition system	ERP on N100 and P200 on Pz and Cz	The research studied the icon design for the military field through ERP analysis.
[84]	Images and words	UI and UX	20	64 electrodes	ERP on N400 (FC1, FC2, FC3, FC4, FCz, C1, C2, C3, C4, Cz, CP1, CP2, CP3, CP4, CPz). ERSP at FCz on time-frequency analysis	The research found that abstract images can be related to similar meanings of words. The research can persuade UX and UI designers to use artistic interaction in more abstract ways.
[108]	Information visualization	UI and UX	15	g.GAMMAcap	Spectral analysis on delta (<4 Hz), theta (4–8), alpha (8–12), beta (12–30), and gamma (30–60)	Visual neuro-biofeedback of spatial visualization can help easily remember numbers and text.
[12]	Mobile phone user experience	UX	8	Neuroscan system and 64 data channels	Delta, theta, gamma, beta, and alpha relative power of topography	The study compared two smartphones with two different user experiences and found that better UX could have higher alpha, delta, and gamma but weaker beta and theta.
[69]	Mobile phone shape	UX	18	23 Ag/AgCl electrodes	Oddball paradigm N100 and N200 ERPs: Frontal lobe (F3, FZ, F4) Central sites (C3, CZ, C4) Prefrontal (FP1, FPZ, FP2)	The study invented the research on using ERP to analyze the visual biofeedback from UX platforms, such as mobile phone shape.
[132]	Navigation interface of mobile game	UI and UX	22	Neuroscan EEG system with 64 Ag/AgCl electrodes	Oddball paradigm N100, P200, and N200 ERPs: Prefrontal (FP1, FPz, FP2) Frontal (F3, Fz, F4) Frontal-central (FC3, FCz, FC4) Central (C3, Cz, C4)	Used ERP to study the navigation of a game environment interface to develop UX on the game market.
[133]	Product description design in online shopping	UI and UX	18	64 electrodes with a Neuroscan SynAmp 2 Amplifier	Observe ERP feedback on P200, N200, and LPP (F1, Fz, F2, FC1, FCz, FC2, C1, Cz, C2, CP1, CPz, CP2, P1, Pz, and P2)	The research utilized ERP to observe negative and positive frame designs on cognitive processing of evaluation biofeedback.
[134]	Road animation	UI and UX	3	Emotiv EPOC	Visual and emotional response (frontal: AF3, AF4; temporal: T7, T8; parietal/occipital: Pz) Topography	The research studied the driving environment comparing rural road city roads, which suits driving game designs or real driving UX research.
[135]	Robotic dance	UX	-	-	-	Although the study did not provide EEG analysis, it created a connection between neuroaesthetics and robotic dance for future brain analysis research. The study created a model of perceiving robotic dance stimuli with brain regions.
[136]	Signs and text	UI	31	Neuroscan SynAmp 2 Amplifier using 64 Ag/AgCl electrodes	ERP on N170, P200, N300, and N400 Frontal (F1, FZ, F2, FC1, FCZ, and FC2) Central-partial area (CP3, CP4, and CPZ) Parietal area (P3, PZ, P4)	The research studied the ERP visual biofeedback on signs and text in UI design.
[137]	Spatial consideration	UX	5	g.USBamp EEG system with g.SAHARA dry electrodes	Steady-state visually evoked potential Oz, O1, O2, POz, PO3, PO4, C1, and C2	The research studied spatial consideration on future UX or UI animation movements.
[138]	Text design	UI and UX	35	fMRI	Rapid serial visual presentation	Comprehensive research to study visual text memory through the ventral visual stream; the mid-fusiform cortex played a role in memorizing long-term visual word forms.
[139]	Traffic interface	UI	36	Emotiv + BCI	Test the mental work, stress, and emotions of reading information on multiple interfaces	The study can be used for future public transportation interface design.
[32]	Time delay of interaction	UX	73	fMRI	Set up three fMRI experiments to compare the results; observe the activated conditions in terms of anterior insular cortex, posterior medial frontal cortex, inferior parietal lobule, and inferior frontal junction	The study analyzed the influence of delay on the UX in a human-computer interaction.
[140]	User character icon design	UI and UX	24	fMRI	Study activations of caudate nucleus, reward circuitry, dorsolateral prefrontal cortex, anterior cingulate cortex, dorsal anterior cingulate cortex, amygdala, etc.	Observed brain region activation through fMRI. The results showed that men prefer online anthropomorphic avatar matching their ethnicity and women avoid interacting with the opposite gender.
[141]	User evaluation	UX	8	QUASAR DSI-24 dry-electrode EEG headset	EEG signals Comparison of subjective evaluations and objective measurements	Tested visual and audio stimuli to compare EEG measurements and subjective evaluations.
[142]	User interface design	UI	13	Neuroscan EEG with 32 electrodes	EEG topography Theta (4–7 Hz), alpha (8–13 Hz), beta (14–30 Hz)	The study used the brain topography to compare two different UI design groups by observing theta, alpha, and beta band activation areas.
[143]	User interface (browsing bar)	UI	-	Eye tracker and Emotiv	Combined eye movement and EEG data	The study combined both eye and brain data to optimize the UI design solutions.
[40]	User interface design (mobile)	UI	9	64-channel elastic electrode cap	Oddball paradigm LPP and N200 were applied Prefrontal (FP1, FPZ, FP2) Frontal (F3, FZ, F4) Central area (C3, CZ, C4)	The study tried to use EEG to analyze visual biofeedback of two different GUI designs.
[91]	User experience concept design	UX	19	BrainProduct actiChamp-32	EEG topography Delta (0.5–3.5 Hz) Theta (4–7 Hz) Alpha (8–12 Hz) Beta (14–25 Hz) Frontal left (FP1, FC9, F3, F7) Frontal right (FC2, FC6) Centrotemporal left (C3, T7) Centrotemporal right (C4, T8) Centroparietal left (CP1, CP5) Centroparietal right (CP2, CP6) Parietotemporal left (P3, P6) Parietotemporal right (P4, P8) Occipital left and right (O1, O2)	The research utilized EEG topography analysis to study open-ended, decision-making, and constrained design problems to improve design performance.
[144]	Visual semantic memory	UX	15	32 channels using an electrode cap (Biosemi)	PE (400–800 ms) and LPN (500–900 ms) ERP electrodes on CP1, Cz, CP2, Pz	The study utilized the high and low visual semantics to explore the visual working memory.
[45]	Visual interface memory	UI and UX	12	Emotiv EPOC+	Test emotion feedback on visual memory performance through stressful or nonstressful environment	The study is related to emotional response on liking of or wanting from the Chatterjee model. It covers data on interest, excitement, engagement, stress, relaxation, and focus. The study also considered the environmental influence on visual memory performance, which helps future designers help people easily memorize visual content of UX and UI designs.
[47]	Virtual game environment	UX	-	Combination of EEG with HTC Vive	P300 BCI	The article summarizes P300 BCI's current and future developments in connection with virtual reality games.
[26]	Website	UI	20	32 sintered Ag/AgCl electrodes	Frontal: F3, Fz, F4 Central: C3, Cz, C4 Parietal: P3, Pz, P4 Occipital: O1, Oz, O2	The research study compared the aesthetic processing between experts and laypersons on judging the beauty of websites.
[145]	Website	UI	16	Neuroscan SynAmp 2 Amplifier with 24 Ag/AgCl electrodes	P200, LPP, and N100 on ERP Frontal (F3, F4) Frontal-central group (FC3, FC4) Central group (C3, C4) Parietal group (P3, P4)	The research suits the Chatterjee model on the emotion stage on liking or wanting by perceiving early vision stimuli.
[146]	Website logo	UI	20	EEG recording caps for 32 channels (CP5, CP1, CP2, CP6, P7, P3, Pz, P4, P8, POz, O1, Oz, O2)	P300 oddball paradigm on ERP analysis	The research defined three specific logo locations on the navigation bar and applied the ERP method to test the best design position of the website logo.

UI, user interface; UX, user experience; ERP, event-related potential; EEG, electroencephalograph; GUI, graphical user interface; fMRI, functional magnetic resonance imaging; BCI, brain-computer interface; VR, virtual reality; SSVEP, steady-state visually evoked potential; RSVP, rapid serial visual presentation.

**Table 6:** SWOT analysis on brain aesthetic processing research on UX and UI designs.

**Strength**	**Weakness**
1. Direct and fast brain reflection of aesthetic preference.	1. Fewer people preferred intracranial experiments.
2. Without ambiguity and dependence on the subjective evaluation results of the users.	2. Clinical grade equipment is not easy to carry and move.
3. Powerful and detailed brain assessment of ERP, time-frequency, topography, and fMRI analysis.	3. Clinical grade equipment does not allow participants to move [147].
4. Interactive wearable market has been in the spotlight.	4. More expensive and time-consuming compared with subjective user evaluations [148].
5. Continuous updates of neurophysiological studies in the world.	5. The data accuracy of wearable BCI on active and reactive BCI experiences cannot compare with that of a clinical grade apparatus.
	6. Environment consideration of processing the experiments such as signal interference and noise [149].

**Opportunity**	**Threat**
1. Neuromarketing is emerging, and neuroscience is ubiquitous in the real world [27, 150].	1. Subjective evaluation needs to become more comprehensive. Otherwise, research starts to be inclined to use electrophysiological data results.
2. Fewer EEG or ERP studies on creative data or information visualization research.	2. Uncomfortable sensor feeling might influence the UX design or user interaction process.
3. Wearable EEG sensor technology can support many interactive platforms, such as Arduino.	3. After users process the practice before the experiment starts, they lose the visual novelty of the real experiment.
4. Possibility of combinations of brain equipment and other electrophysiological apparatus.	
5. More comfortable and precise wearable interactive technology and devices are being invented.	

SWOT, strengths, weaknesses, opportunities, and threats; UX, user experience; UI, user interface; EEG, electroencephalograph; ERP, event-related potential; BCI, brain-computer interface; fMRI, functional magnetic resonance imaging.

## Data Availability

No data were used to support this study.
